# Assessment of disaster preparedness and related impact factors among emergency nurses in tertiary hospitals: descriptive cross-sectional study from Henan Province of China

**DOI:** 10.3389/fpubh.2023.1093959

**Published:** 2023-05-04

**Authors:** Jiange Zhang, Lei Yang, Xue Cao, Ying Ren, Xu Han, Shuting Zang, Fangfang Cai, Lijun Xu, Lijie Qin, Peirong Zhang, Yanwei Cheng

**Affiliations:** ^1^Department of Emergency, Henan Provincial People's Hospital, People's Hospital of Zhengzhou University, People's Hospital of Henan University, Zhengzhou, China; ^2^Henan Provincial Key Medicine Laboratory of Nursing, Henan Provincial People's Hospital, Zhengzhou, China; ^3^Department of Rheumatology and Immunology, Henan Provincial People's Hospital, People's Hospital of Zhengzhou University, People's Hospital of Henan University, Zhengzhou, China; ^4^Henan University School of Nursing and Health, Zhengzhou, China

**Keywords:** disaster preparedness, emergency nurses, tertiary hospitals, descriptive cross-sectional study, disaster nursing specialist nurse training

## Abstract

**Background:**

The aim of this study was to investigate the current state of disaster preparedness and to determine associated factors among emergency nurses from tertiary hospitals in Henan Province of China.

**Methods:**

This multicenter descriptive cross-sectional study was conducted with emergency nurses from 48 tertiary hospitals in Henan Province of China between September 7, 2022–September 27, 2022. Data were collected through a self-designeds online questionnaire using the mainland China version of the Disaster Preparedness Evaluation Tool (DPET-MC). Descriptive analysis and multiple linear regression analysis were used to evaluate disaster preparedness and to determine factors affecting disaster preparedness, respectively.

**Results:**

A total of 265 emergency nurses in this study displayed a moderate level of disaster preparedness with a mean item score of 4.24 out 6.0 on the DPET-MC questionnaire. Among the five dimensions of the DPET-MC, the mean item score for pre-disaster awareness was highest (5.17 ± 0.77), while that for disaster management (3.68 ± 1.36) was the lowest. Female gender (B = −9.638, *p* = 0.046) and married status (B = −8.618, *p* = 0.038) were negatively correlated with the levels of disaster preparedness. Five factors positively correlated with the levels of disaster preparedness included having attended in the theoretical knowledge training of disaster nursing since work (B = 8.937, *p* = 0.043), having experienced the disaster response (B = 8.280, *p* = 0.036), having participated in the disaster rescue simulation exercise (B = 8.929, *p* = 0.039), having participated in the disaster relief training (B = 11.515, *p* = 0.025), as well as having participated in the training of disaster nursing specialist nurse (B = 16.101, *p* = 0.002). The explanatory power of these factors was 26.5%.

**Conclusion:**

Emergency nurses in Henan Province of China need more education in all areas of disaster preparedness, especially disaster management, which needs to be incorporated into nursing education, including formal and ongoing education. Besides, blended learning approach with simulation-based training and disaster nursing specialist nurse training should be considered as novel ways to improve disaster preparedness for emergency nurses in mainland China.

## Introduction

Continuously occurring yet unpredictable disasters remain a serious threat to global public health and property (1). In mainland China, the main forces of disaster relief contain national emergency medical rescue teams, private rescue organizations and healthcare facilities (2). Among them, healthcare facilities are regarded as vitally important emergency response resources because they are central to providing timely and good quality healthcare services for the injuries (3). With the increasing disaster events, healthcare facilities are expected to enhance frontline force to cope with disaster disruptions. As the major frontline workforce in disaster relief, nurses, especially emergency nurses, are of particular importance. When a disaster strikes, emergency nurses can detect threats, provide direct nursing care to patients, manage healthcare teams and facilities, reduce or eliminate injuries and deaths, develop healthcare policies, and work in collaboration with other organizations (4, 5). In addition, increasing studies have shown that disaster preparedness is a crucial factor in disaster response that can reduce the damaging effects of emergencies and disasters (6, 7). Therefore, mobilizing emergency nurses to improve disaster preparedness capability has become a pivotal strategy to enhance the capability of healthcare facilities to respond to different types of disasters.

Disaster preparedness is defined as the reserve of knowledge and capacity to effectively respond to disasters (8). More and more attention has been paid to investigate disaster preparedness of emergency nurses in recent years. At present, most of studies abroad on disaster preparedness of emergency nurses clearly reported a weak-to-average or a low-to-moderate level of disaster preparedness and concluded that emergency nursers remained inadequately prepared on all domains of disaster nursing competencies (9–11). A recent study in Guangdong Province of China reported that emergency nurses had a moderate level of disaster preparedness, and addressed that hospitals and nurse managers should carry out interdisciplinary and multidisciplinary cooperation to improve emergency nurses' disaster preparedness (12). Assessing the extent of disaster preparedness among emergency nurses and identifying associated influencing factors can contribute to refine the practical strategies for enhancing their disaster preparedness capability. However, current study on disaster preparedness among emergency nurses in China is extremely limited.

As the second largest economy in the world, China became the third most disaster-prone country in Asia in 2021, after Indonesia and India (13). Due to the complex topography and high density of population, China's Henan Province are constantly exposed to a variety of unexpected disasters including geological disasters, fire disasters, traffic disasters, meteorological disasters, and infectious diseases, thus always suffer catastrophic consequences, such as human casualties and property damage. For instance, in July 2021, Henan Province was struck by an extraordinarily heavy rainstorm that caused severe flooding, resulting in 352 deaths, 14.5 million population affected, and a cost of $16.5 billion (13). This sudden mega-disaster not only posed unprecedented challenges to disaster emergency management system but also exposed the poor disaster preparedness among municipal government, healthcare facilities and medical personnel (14). Considering the vital role of emergency nurses on the frontline of disasters response, the disaster preparedness of emergency nurses in Henan Province is of great concern. However, little is known regarding the prevalence of disaster preparedness among emergency nurses in Henan Province of China, which may impede further improvement of the quality and efficiency of disaster relief provided by healthcare facilities.

Thus, the present study aimed to investigate the current state of disaster preparedness among emergency nurses from tertiary hospitals in Henan Province of China, and to determine key related factors affecting disaster preparedness to improve disaster preparedness capability.

## Methods

### Study design, setting, and participants

This descriptive cross-sectional study was conducted with emergency nurses from 48 tertiary hospitals, which all have >1,000 beds and spread out across 17 cities in Henan Province, China, from September 7, 2022 to September 27, 2022. To be eligible to participate, nurses were required to be (1) certified registered and full-time nurses; (2) formally working for a minimum of 1 year in emergency department; (3) active during the data collection stage; (4) sufficiently agree to participate. Nurses with older than 50 years were excluded from the study because they were basically no longer frontliners in the hospital.

### Ethical consideration

The Ethics Committee of Henan Provincial People's Hospital approved the study. All participants were provided a description of the study and informed that participation was voluntary, and anonymity assured. In addition, participants were informed that they could withdraw from the study at any time.

### Questionnaire

The online questionnaire consists of two parts. The first part focused on the demographic and work-related characteristics, and the second part focused on the assessment of disaster preparedness. All items of the demographic and work-related questionnaire were shown in Table 1. The Mainland China version of the Disaster Preparedness Evaluation Tool (DPET-MC) scale, a modified version of the DPET (15), is constructed by Yibing Tan (16) (as shown in Supplementary material) and is a validated instrument to assess disaster preparedness of emergency nurses in mainland China. The DPET-MC scale consists of 34 items within five subscales: pre-disaster awareness (3 items), pre-disaster knowledge (8 items), disaster management (10 items), knowledge and skills in the workplace (6 items), and post-disaster knowledge and skills (7 items). The Cronbach's alpha coefficients of the DPET-MC are 0.79, 0.88, 0.94, 0.90, 0.96 and 0.97 for the five dimensions and the overall scale, respectively. The 34 items were assessed using a 6-point Likert scale (1= “strongly disagree”; 6 = “strongly agree”). The total score ranges from 34 to 204, with a higher score indicating a higher level of disaster preparedness. The DPET-MC was used in our study with permission from Yibing Tan (16).

**Table 1:** Demographic and work-related characteristics of emergency nurses (*N* = 265).

**Variables**	***n***	**%**
**Age**		
20–30 years	100	37.7
31–40 years	151	57.0
41–50 years	14	5.3
**Gender**		
Male	55	20.8
Female	210	79.2
Marital status		
Married	187	70.6
Single	78	29.4
**Educational level**		
Post diploma	15	5.7
Bachelor's degree	233	87.9
Master's or PhD degree	17	6.4
**Health condition**		
Fitness	156	58.9
Good	73	27.5
Average	32	12.1
Poor	4	1.5
**Work duration**		
≤5 years	85	32.1
6~10 years	92	34.7
11~15 years	58	21.9
≥16 years	30	11.3
**Professional title**		
Primary title	106	40.0
Intermediate title	157	59.2
Senior title	2	0.8
**Having attended disaster nursing courses at school**		
Yes	146	55.1
No	119	44.9
**Having participated in the theoretical knowledge training of disaster nursing since work**		
Yes	158	59.6
No	107	40.4
**Having participated in the disaster rescue simulation exercise**		
Yes	133	50.2
No	132	49.8
**Having participated in the disaster relief training**		
Yes	221	83.4
No	44	16.6
**Having participated in the training of disaster nursing specialist nurse**		
Yes	52	19.6
No	213	80.4
**Having experienced the disaster response**		
Yes	150	56.6
No	115	43.4
**Willingness to participate in the disaster relief mission**		
Yes	245	92.5
No	20	7.5

### Data collection

Data were collected through the aforementioned online questionnaire via a software of Questionnaire Star (17), which was conducted through WeChat software based on the smartphone. Participants who agreed to participate in the study were required to open the questionnaire and submit it only after completing all questions, so it was impossible to submit an incomplete answer. To avoid repeated access and completion of the questionnaire, an internet provider number was used for access restriction. Completed questionnaires were collected automatically and exported to an Excel file after the respondents answered the questions.

### Data analysis

Descriptive statistics were performed to summarize the demographic and some work-related characteristics of the entire study population. Continuous data were expressed as mean ± standard deviation (M ± SD), whereas categorical data were presented as an absolute number and percentage. Independent sample *t*-tests and one-way analysis of variance (ANOVA) were carried out to determine the differences and associations between demographic and work-related variables and disaster preparedness. Variables with *p* < 0.10 were included in the multivariate analysis. The multiple linear regression model was performed to identify salient variables associated with disaster preparedness among demographic and work-related factors. All *p*-values are two-sided. *p* < 0.05 was considered as statistically significant. All analyses were conducted using SPSS statistical software version 25.0 (IBM Corporation, Armonk, NY).

## Results

### Demographic and work-related characteristics

Of the 365 questionnaires handed out, 280 were returned, resulting in an 76.7% response rate. 15 were excluded due to various combinations of age (>50 years), work duration (<1 year), and nonregistered nurses. Finally, a total of 265 questionnaires were analyzed in this study.

Demographic and work-related characteristics are summarized in Table 1. Of the included participants with age from 20 to 48 years, 151 (57.0%) were in the 30 to 40 age group. 210 (79.2%) were female. 187 (70.6%) were married and 233 (87.9%) had a bachelor's degree. 85 (32.1%) respondents had a work duration of <5 years. More than half of respondents had attended disaster nursing courses, trainings and exercise, while only 52 (19.6%) had participated in the training of disaster nursing specialist nurse. 150 (56.6%) had the disaster response experience. Notably, 20 (7.5%) expressed less willing to participate in any disaster relief mission.

### Disaster preparedness among emergency nurses

The total score range, mean ± SD score, mean item score, skewness, and kurtosis for the DPET-MC scale and for its five dimensions are presented in Table 2. The total scale score ranged from 62 to 204, with a mean score of 144.19 (SD = 34.00) and a mean item score of 4.24 (SD = 1.00) out 6.0. The dimensions with the highest and lowest mean item scores were pre-disaster awareness (5.17 ± 0.77) and disaster management (3.68 ± 1.36), respectively.

**Table 2:** Emergency nurses' disaster preparedness' scores (*N* = 265).

	**Total score range**	**Total score**	**Item score**	**Skewness**	**Kurtosis**
		**Mean (SD** ***)***	**Mean (SD)**		
Total scale	62–204	144.19 (34.00)	4.24 (1.00)	0.067	−0.666
Pre-disaster awareness	9–18	15.51 (2.32)	5.17 (0.77)	−0.529	−0.714
Pre-disaster knowledge	16–48	36.86 (7.65)	4.61 (0.96)	−0.097	−0.754
Knowledge and skills in the workplace	6–36	26.09 (6.54)	4.35 (1.09)	−0.357	−0.123
Post-disaster skills and knowledge	7–42	28.97 (8.26)	4.14 (1.18)	−0.339	−0.133
Disaster management	10–60	36.76 (13.63)	3.68 (1.36)	0.081	−0.805

### Univariate analysis of factors affecting disaster preparedness

Independent sample *t*-tests and one-way analysis of variance revealed that nurses with male gender, single and fitness health condition, those who had attended disaster nursing courses at school, those who had participated in the theoretical knowledge training of disaster nursing since work, those who had participated in the disaster rescue simulation exercise and the disaster relief training, those who had participated in the training of disaster nursing specialist nurse, and those who had experienced the disaster response were more likely to report higher item scores of the DPET-MC, indicating better disaster preparedness. Nevertheless, factors such as age, educational level, work duration, professional title, and willingness to participate in the disaster relief mission showed no correlation with disaster preparedness (Table 3).

**Table 3:** Univariate analysis of factors affecting disaster preparedness (*N* = 265).

	**Pre-disaster awareness**	**Pre-disaster knowledge**	**Disaster management**	**Knowledge and skills in the workplace**	**Post-disaster skills and knowledge**	**Item score for the DPET-MC**
**Mean (SD)**						
**Age**						
20–30 years	5.12 (0.77)	4.58 (0.98)	3.81 (1.37)	4.37 (1.02)	4.13 (1.15)	4.27 (1.01)
31–40 years	5.17 (0.79)	4.58 (0.95)	3.53 (1.36)	4.29 (1.14)	4.09 (1.22)	4.17 (1.01)
41–50 years	5.52 (0.62)	5.10 (0.72)	4.34 (1.06)	4.76 (0.91)	4.64 (0.91)	4.76 (0.71)
F	1.659	1.960	3.143	1.228	1.389	2.321
*p* (ANOVA)	0.192	0.143	0.045	0.295	0.251	0.098
**Gender**						
Male	5.25 (0.80)	4.93 (0.93)	4.25 (1.22)	4.85 (0.86)	4.69 (1.01)	4.69 (0.89)
Female	5.15 (0.77)	4.52 (0.95)	3.53 (1.36)	4.22 (1.11)	4.00 (1.18)	4.12 (1.00)
T	0.913	2.800	3.592	3.901	3.971	3.860
*p* (t-test)	0.362	0.005	0.000	0.000	0.000	0.000
**Marital status**						
Single	5.22 (0.78)	4.68 (0.97)	3.78 (1.39)	4.44 (1.12)	4.26 (1.21)	4.33 (1.02)
Married	5.05 (0.76)	4.44 (0.91)	3.43 (1.27)	4.12 (0.99)	3.85 (1.07)	4.02 (0.91)
T	1.677	1.877	1.873	2.245	2.618	2.359
*p* (t-test)	0.095	0.062	0.062	0.026	0.009	0.019
**Educational level**						
Post diploma	5.11 (0.79)	4.72 (1.03)	3.63 (1.44)	4.40 (0.88)	4.27 (1.01)	4.28 (0.98)
Post diploma	5.18 (0.78)	4.62 (0.96)	3.70 (1.37)	4.36 (1.11)	4.14 (1.20)	4.25 (1.01)
Master1.01)alevele DPE	5.04 (0.60)	4.36 (0.86)	3.46 (1.20)	4.12 (1.00)	4.07 (1.03)	4.06 (0.89)
F	3.266	3.992	4.911	10.954	5.715	7.161
*p* (ANOVA)	0.727	0.508	0.781	0.661	0.887	0.720
**Health condition**						
Fitness	5.75 (0.32)	5.22 (1.06)	4.33 (1.58)	4.75 (1.66)	4.36 (1.52)	4.74 (1.20)
Good	5.22 (0.78)	4.75 (0.97)	3.83 (1.33)	4.49 (1.05)	4.28 (1.18)	4.38 (0.99)
Average	5.11 (0.73)	4.41 (0.87)	3.43 (1.31)	4.25 (1.31)	3.99 (1.28)	4.03 (1.12)
Poor	5.00 (0.84)	4.30 (0.97)	3.43 (1.53)	4.08 (1.00)	3.88 (1.08)	4.01 (0.93)
F	1.629	4.004	2.086	2.677	2.224	3.073
*p* (ANOVA)	0.183	0.008	0.102	0.048	0.086	0.028
**Work duration**						
≤5 years	5.05 (0.73)	4.56 (0.86)	3.68 (1.31)	4.15 (1.04)	3.99 (1.11)	4.16 (0.96)
6–10 years	5.22 (0.81)	4.61 (1.05)	3.72 (1.46)	4.41 (1.14)	4.19 (1.28)	4.28 (1.08)
11–15 years	5.17 (0.79)	4.58 (0.98)	3.56 (1.31)	4.50 (1.08)	4.22 (1.12)	4.24 (0.97)
≥16 years	5.34 (0.70)	4.80 (0.89)	3.73 (1.37)	4.41 (1.09)	4.26 (1.19)	4.35 (0.98)
F	1.277	0.463	0.187	1.402	0.701	0.363
*p* (ANOVA)	0.283	0.708	0.905	0.243	0.552	0.780
**Professional title**						
Primary title	5.15 (0.78)	4.65 (0.95)	3.82 (1.36)	4.38 (1.03)	4.15 1.18)	4.30 (0.99)
Intermediate title	5.18 (0.77)	4.58 (0.97)	3.58 (1.37)	4.32 (1.13)	4.12 (1.19)	4.20 (1.02)
Senior title	5.33 (0.00)	4.75 (0.35)	4.25 (1.06)	4.75 (0.35)	4.86 (0.20)	4.68 (0.50)
F	0.091	0.223	1.161	0.245	0.387	0.522
*p* (ANOVA)	0.913	0.800	0.315	0.783	0.680	0.594
**Having attended disaster nursing courses at school**						
Yes	5.14 (0.78)	4.74 (0.85)	3.89 (1.21)	4.45 (0.98)	4.30 (1.04)	4.38 (0.89)
No	5.20 (0.77)	4.45 (1.06)	3.41 (1.49)	4.22 (1.21)	3.94 (1.30)	4.07 (1.10)
T	−0.659	2.413	2.804	1.722	2.459	2.55
*p* (t-test)	0.511	0.017	0.005	0.086	0.015	0.011
**Having participated in the theoretical knowledge training of disaster nursing since work**						
Yes	5.19 (0.77)	4.80 (0.90)	3.98 (1.25)	4.58 (0.96)	4.43 (1.05)	4.48 (0.91)
No	5.15 (0.78)	4.33 (0.98)	3.23 (1.41)	4.01 (1.18)	3.71 (1.24)	3.90 (1.03)
T	0.405	3.985	4.508	4.348	5.071	4.822
*p* (t-test)	0.686	0.000	0.000	0.000	0.000	0.000
**Having participated in the disaster rescue simulation exercise**						
Yes	5.31 (0.73)	4.89 (0.91)	4.08 (1.26)	4.65 (1.05)	4.51 (1.13)	4.57 (0.94)
No	5.03 (0.79)	4.34 (0.92)	3.27 (1.35)	4.04 (1.05)	3.76 (1.12)	3.91 (0.95)
T	3.083	5.006	5.059	4.77	5.436	5.667
*p* (t-test)	0.002	0.000	0.000	0.000	0.000	0.000
**Having participated in the disaster relief training**						
Yes	5.23 (0.75)	4.66 (0.96)	3.78 (1.36)	4.46 (1.03)	4.23 (1.15)	4.33 (0.98)
No	4.86 (0.80)	4.33 (0.91)	3.15 (1.26)	3.78 (1.19)	3.66 (1.23)	3.80 (0.97)
T	2.919	2.102	2.830	3.915	2.976	3.291
*p* (t-test)	0.004	0.036	0.005	0.000	0.003	0.001
**Having participated in the training of disaster nursing specialist nurse**						
Yes	5.41 (0.75)	5.08 (0.84)	4.51 (1.08)	4.95 (0.87)	4.90 (0.91)	4.88 (0.82)
No	5.11 (0.77)	4.49 (0.95)	3.47 (1.35)	4.20 (1.09)	3.95 (1.17)	4.08 (0.98)
T	2.529	4.082	5.152	4.624	5.434	5.414
*p* (t-test)	0.012	0.000	0.000	0.000	0.000	0.000
**Having experienced the disaster response**						
Yes	5.26 (0.74)	4.79 (0.98)	3.96 (0.37)	4.60 (1.10)	4.39 (1.21)	4.47 (1.02)
No	5.06 (0.80)	4.37 (0.87)	3.30 (1.26)	4.02 (0.99)	3.81 (1.06)	3.94 (0.90)
T	2.076	3.626	4.039	4.421	4.087	4.449
*p* (t-test)	0.039	0.000	0.000	0.000	0.000	0.000
**Willingness to participate in a disaster relief mission**						
Yes	5.16 (0.77)	4.61 (0.96)	3.67 (0.38)	4.36 (1.10)	4.15 (1.20)	4.24 (1.02)
No	5.28 (0.76)	4.53 (0.90)	3.72 (1.20)	4.26 (0.92)	4.03 (0.88)	4.20 (0.80)
T	−0.683	0.401	−0.132	0.384	0.433	0.170
*p* (t-test)	0.495	0.689	0.895	0.701	0.665	0.865

### Multiple linear regression analysis of factors affecting disaster preparedness

Ten variables with *p* < 0.10 obtained in univariate analysis were entered into a stepwise multiple linear regression model with disaster preparedness score as the dependent variable. The final model had an *R*^2^ of 0.265 and adjusted *R*^2^ of 0.233 (F = 8.284, *p* < 0.001), by which 23.3% of variation in disaster preparedness score could be explained (Table 4). Results showed that female gender (B = −9.638, t = −2.003, *p* = 0.046) and married status (B = −8.618, t = −2.086, *p* = 0.038) had a negative correlation with disaster preparedness. While the rest of five variables were positively correlated with disaster preparedness, including having participated in the theoretical knowledge training of disaster nursing since work (B = 8.937, t = 2.039, *p* = 0.043), having experienced the disaster response (B = 8.280, t = 2.111, *p* = 0.036), having participated in the disaster rescue simulation exercise (B = 8.929, t = 2.080, *p* = 0.039), having participated in the disaster relief training (B = 11.515, t = 2.248, *p* = 0.025), and having participated in the training of disaster nursing specialist nurse (B = 16.101, t = 3.125, *p* = 0.002).

**Table 4:** Multiple linear regression analysis of factors associated with emergency nurses' disaster preparedness scores (*N* = 265).

**Model**	**B**	**SE**	**Beta**	***t***	***P*-Value**
(constant)	94.783	19.567	-	4.844	0.000
Gender (reference = Male)	−9.638	4.813	−0.115	−2.003	0.046
Marital status (reference = Single)	−8.618	4.132	−0.116	−2.086	0.038
Having participated in the theoretical knowledge training of disaster nursing since work (reference = No)	8.937	4.384	0.129	2.039	0.043
Having experienced the disaster response (reference = No)	8.280	3.922	0.121	2.111	0.036
Having participated in the disaster rescue simulation exercise (reference = No)	8.929	4.293	0.132	2.080	0.039
Having participated in the disaster relief training (reference = No)	11.515	5.122	0.126	2.248	0.025
Having participated in the training of disaster nursing specialist nurse (reference = No)	16.101	5.153	0.188	3.125	0.002

*R*^2^= 0.265; Adjusted *R*^2^= 0.233; *F* = 8.284; *p* < 0.001.

## Discussion

As the major frontline force in combating against disasters, emergency nurses must be adequately prepared to cope with them. The present study reported that emergency nurses in Henan Province perceived they had a moderate level of disaster preparedness, and determined seven factors affecting disaster preparedness among emergency nurses. The results are expected to provide valuable information for hospital administrators and nurse educators on how to improve the disaster preparedness capability and capacity of emergency nurses.

Multiple cross-sectional surveys of disaster preparedness among clinical nurses in various countries reported a same outcome that nurses had overall a low-to-moderate level of disasters preparedness using varied scales (5, 18–24). Studies in Hong Kong, Taiwan and Changsha also found that nurses were inadequately prepared for disaster response (25–27). However, nurses in Henan Province perceived that they had a moderate level of disaster preparedness using the DPET-MC questionnaire, and seemed to present a higher level of disaster preparedness in comparison with nurses in other cities/countries. The reason for this discrepancy might be that the respondents of previous studies were nurses, whereas those of this study were emergency nurses, who are first responders in disaster scenes and have more sufficient experience and knowledge about how to manage different emergency situation compared to nurses in other departments (28–30). Besides, this discrepancy might also be at least partially caused by the different instruments assessing disaster preparedness. The DPET-MC with internal consistency reliability and split-half reliability in this study had fewer items than the original DPET scale and could save time for emergency nurses in a fast-paced work environment (16). It therefore is targeted at emergency nurses in mainland China and is more suitable for our study. Of note, in the recent study among emergency nurses in Guangdong Province, Wang et al. also used the DPET-MC questionnaire and reported that the perceived disaster preparedness of emergency nurses was at a moderate level (12). At present, there have been only limited studies to investigate the disaster preparedness among Chinese emergency nurses. These findings might contribute to a comprehensive understanding of the level of disaster preparedness among emergency nurses in China.

Compared with the prior systematic review of literature that reported a general lack of disaster awareness among nurses abroad (1), it is worth noting that emergency nurses in the present study displayed the highest mean item score for pre-disaster awareness. This finding is congruent with another Chinese study by Wang et al. (12). The reasons for this gap might be associated with the high incidence of disaster events in China and that almost all Chinese emergency nurses experienced in fighting against COVID-19 pandemic. However, the mean item score for disaster management in the present study was the lowest, which is consistent with the results acquired from many previous studies (12, 24), reflecting that disaster management is the most challenging dimension in disaster preparedness and the disaster management ability of emergency nurses needs to be improved most urgently. Therefore, it is necessary for nursing education to incorporate the disaster management education into formal and ongoing education.

While emergency nurses in Henan Province were not fully prepared for disasters, in line with previous studies, several factors may improve disaster preparedness. Prior studies have established the associations between disaster preparedness and gender, as well as marital status (12, 31). Likewise, our study identified that both female gender and married status were negatively correlated with perceived levels of disaster preparedness. Reasons might be that female and married nurses have weaker physical and psychological qualities to undertake rescue work. Thus, hospitals should provide more physical, technical, and psychological education and training for female and married nurses to improve the ability of disaster relief, as well as more humanistic care for married nurses to avoid excessive worries about their families.

Previous studies have found experience is the basis of preparedness, and experienced nurses are better able to provide care in emergency situations than less experienced nurses (32). The present study also showed that having experienced the disaster response were positively correlated with disaster preparedness. It is recommended that participation by emergency nurses in real or mock drill experience is a useful adjunct for promoting disaster preparedness. In addition, three other factors positively affecting the disaster preparedness level of emergency nurses were reported in the present study, including having participated in the theoretical knowledge training of disaster nursing since work, having participated in the disaster rescue simulation exercise, and having participated in the disaster relief training. The above three factors can not only exert a pivotal impact on disaster preparedness (22, 33, 34), but also determine the education framework for emergency nurses, suggesting that the teaching approach and strategy for emergency nurse training should be refined. Blended learning is a combination of remote and face-to-face and synchronous and asynchronous teaching methods (35). Prior studies have demonstrated that blended learning is one of the most effective teaching methods in disaster medicine, via multiple teaching strategies it provides the opportunity to teach broad aspects of knowledge and skills (36). In addition, simulation-based training, such as tabletop exercises, drill, functional and full-scale exercise, and virtual reality simulation exercises, has become a realistic and effective approach to prepare the first responders for disaster management and increase the learner knowledge, enhance self-confidence and refine the skills (37–39). Therefore, blended learning approach with simulation-based training would be useful and feasible for disaster nursing education.

In the present study, the factor of having participated in the training of disaster nursing specialist nurse was also significantly and positively correlated with disaster preparedness, and it had never been explored before. Disaster nursing specialist nurse training is a unique disaster nursing model in China, which was launched by the Chinese Nursing Association in 2015 (40). International studies have found that more than half of the nurses had not received any education or training on disasters (5, 41–43). In this study, nearly half of participants lacked the disaster-related education and various training and disaster response experience. Notably, the training of disaster nursing specialist nurse exactly covers basic theory of disaster nursing, professional skills of disaster relief, virtual reality disaster simulation exercises, etc. (44). Although China's disaster nursing is still in its infancy and exploratory stage (45), this finding highlighted that disaster nursing specialist nurse training might be a novel and effective way to enhance disaster preparedness for Chinese emergency nurses.

The limitations of this study are as follows: firstly, given the largely perception-based and does not rely on objective data, the results regarding reflecting actual disaster responses need to be interpreted with caution. However, the findings provided a baseline that can guide planning for continuing disaster education programs and training activities to meet the needs of emergency nurses from mainland China. Secondly, the response rate was not particularly high due to the lack of direct communication between the researcher and participants, which could affect the generalizability of the results. However, the response rate is similar to prior studies (31, 46, 47) and may be applied to similar settings and contexts. Thirdly, causal relationships could not be established in the cross-sectional study. Further studies could collect time-based data to explore causal relationships between disaster preparedness and associated factors. In addition, the variables included in this study might be not comprehensive enough. More relevant factors should be considered in future studies, such as intensive care experience, military hospital, and income.

## Conclusion

To our best knowledge, this is the first study on investigating the levels of disaster preparedness among emergency nurses in Henan Province, China. It is clear from the present study that emergency nurses had a moderate level of disaster preparedness, and their disaster management ability needs to be improved most urgently. Importantly, seven factors affecting disaster preparedness were determined, which offered practical strategies for improvement of disaster preparedness among emergency nurses. Optimizing and detailing these strategies will be an interesting issue that is worth pursuing in the future.

## Data availability statement

The original contributions presented in the study are included in the article/Supplementary material, further inquiries can be directed to the corresponding authors.

## Ethics statement

The Ethics Committee of Henan Provincial People's Hospital approved the study. All participants were provided a description of the study and informed that participation was voluntary, and anonymity assured. In addition, participants were informed that they could withdraw from the study at any time.

## Author contributions

Conceived and designed the study: YC and PZ. Collected and analyzed data: JZ and XC. Designed the tables: LY, FC, and YR. Wrote the first draft of this article: JZ and YC. Revised the manuscript for important intellectual content: SZ, LQ, XH, LX, and PZ. All authors approved the final version.
