# Supplementary material for: Assessment of disaster preparedness and related impact factors among emergency nurses in tertiary hospitals: descriptive cross-sectional study from Henan Province of China
Source: Front Public Health. 2023 May 4;11:1093959. doi: 10.3389/fpubh.2023.1093959 (PMC10192630; doi:10.3389/fpubh.2023.1093959)
Supplement: Supplementary file 1 [file Table_1.DOCX]

**The Chinese mainland version of the Disaster Preparedness Evaluation Tool (DPET-CM).**

| Item Description | 1 | 2 | 3 | 4 | 5 | 6 |
| --- | --- | --- | --- | --- | --- | --- |
| **Pre-disaster awareness** | | | | | | |
| I would be interested in educational classes on disaster preparedness that relate specifically to my community situation. |  |  |  |  |  |  |
| I know the limits of my knowledge, skills, and authority as an NP to act in disaster situations. |  |  |  |  |  |  |
| I am aware of potential vulnerabilities in my community (e.g. earthquake, floods, terror). |  |  |  |  |  |  |
| **Pre-disaster knowledge** | | | | | | |
| I find that the research literature on disaster preparedness is understandable. |  |  |  |  |  |  |
| I have a list of contacts in the medical or health community in which I practice. I know referral contacts in case of a disaster situation (eg, health department). |  |  |  |  |  |  |
| I read journal articles related to disaster preparedness. |  |  |  |  |  |  |
| I know where to find relevant research or information related to disaster preparedness and management to fill in gaps in my knowledge. |  |  |  |  |  |  |
| I find that the research literature on disaster preparedness and management is easily accessible. |  |  |  |  |  |  |
| In case of a disaster situation, I think that there is sufficient support from local officials on the county or state level. |  |  |  |  |  |  |
| I know who to contact (chain of command) in disaster situations in my community. |  |  |  |  |  |  |
| I am aware of classes about disaster preparedness and management that are offered in my workplace, the university, or community. |  |  |  |  |  |  |
| **Disaster management** | | | | | | |
| I have participated in emergency planning for disaster situations in my community. |  |  |  |  |  |  |
| I participate/have participated in creating new guidelines or emergency plans or in lobbying for improvements on the local or national level. |  |  |  |  |  |  |
| In case of a bioterrorism/biological attack, I know how to use personal protective equipment. |  |  |  |  |  |  |
| In case of a bioterrorism/biological attack, I know how to execute decontamination procedures. |  |  |  |  |  |  |
| I consider myself prepared for the management of disasters. |  |  |  |  |  |  |
| I would be considered a leadership figure in my community in a disaster situation. |  |  |  |  |  |  |
| I have personal/family emergency plans in place for disaster situations. |  |  |  |  |  |  |
| I am familiar with psychological interventions, behavioral therapy, cognitive strategies, support groups and incident debriefing for patients who experience emotional or physical trauma. |  |  |  |  |  |  |
| I am able to describe my role in the response phase of a disaster in the context of my workplace, the general public, media, and personal contacts. |  |  |  |  |  |  |
| I am familiar with the organizational logistics and roles among local, state, and federal agencies in disaster response situations.  **Knowledge and skills in the workplace** |  |  |  |  |  |  |
| I am familiar with accepted triage principles used in disaster situations. |  |  |  |  |  |  |
| In a case of bioterrorism/biological attack, I know how to perform isolation procedures so that I minimize the risks for community exposure. |  |  |  |  |  |  |
| Some research has shown that NPs feel constrained by medical malpractice concerns or license restrictions in terms of responding to disasters. This constraint applies to me too. |  |  |  |  |  |  |
| I feel reasonably confident I can treat patients independently without supervision of a physician in a disaster situation. |  |  |  |  |  |  |
| I can identify possible indicators of mass exposure evidenced by a clustering of patients with similar symptoms. |  |  |  |  |  |  |
| I would feel confident working as a triage NP and setting up temporary clinics in disaster situations.  **Post-disaster knowledge and skills** |  |  |  |  |  |  |
| I would feel reasonably confident providing patient education on stress and abnormal functioning related to trauma. |  |  |  |  |  |  |
| I feel reasonably confident discerning deviations in health assessments indicating potential exposure to biological agents. |  |  |  |  |  |  |
| I am familiar with what the scope of my role would be in a post-disaster situation. |  |  |  |  |  |  |
| I am able to discern signs and symptoms of acute stress disorder and post-traumatic stress disorders (PTSD). |  |  |  |  |  |  |
| I am comfortable providing education on coping skills and training for patients who experience traumatic situations. |  |  |  |  |  |  |
| I am comfortable managing (treating, evaluating) emotional outcomes for acute stress disorder or PTSD. |  |  |  |  |  |  |
| I am familiar with how to perform focused health assessment for PTSD. |  |  |  |  |  |  |

The score rule: “1”= “strongly disagree”; “2”= “disagree”; “3”= “partly disagree”; “4”= “partly agree”; “5”= “agree”; “6”= “strongly agree”.

Five factors explained 64.06% of the total variance; the fit indices were: $x^{2}$/DF=1.978, RMSEA=0.071, IFI=0.907, TLI=0.90, CFI=0.90.
